# Supplementary material for: SARS-CoV-2 Accessory Protein Orf7b Induces Lung Injury via c-Myc Mediated Apoptosis and Ferroptosis
Source: Int J Mol Sci. 2024 Jan 18;25(2):1157. doi: 10.3390/ijms25021157 (PMC10816122; doi:10.3390/ijms25021157)
Supplement: Supplementary file 1 [file ijms-25-01157-s001.zip › ijms-2801855-supplementary figures.pdf]

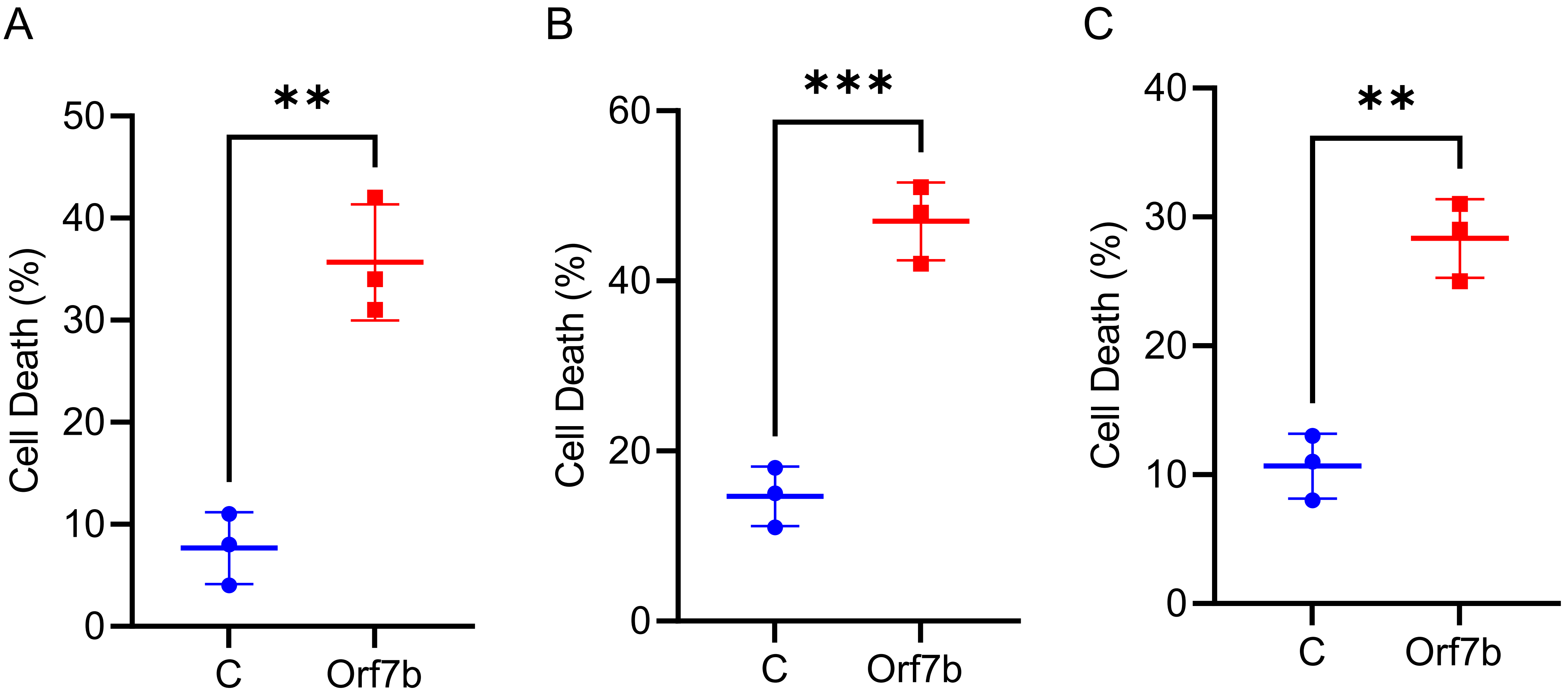

Supplementary Fig S1. Results of LDH assay for Beas 2B cells (A), A549 cells (B) and primary cells (HSAEC)(C) suggested Orf7b induced cytotoxicity in all the three the cell lines. C: control group, Orf7b: Orf7b overexpressed group. **: P ≤ 0.01, ***: P ≤ 0.001.

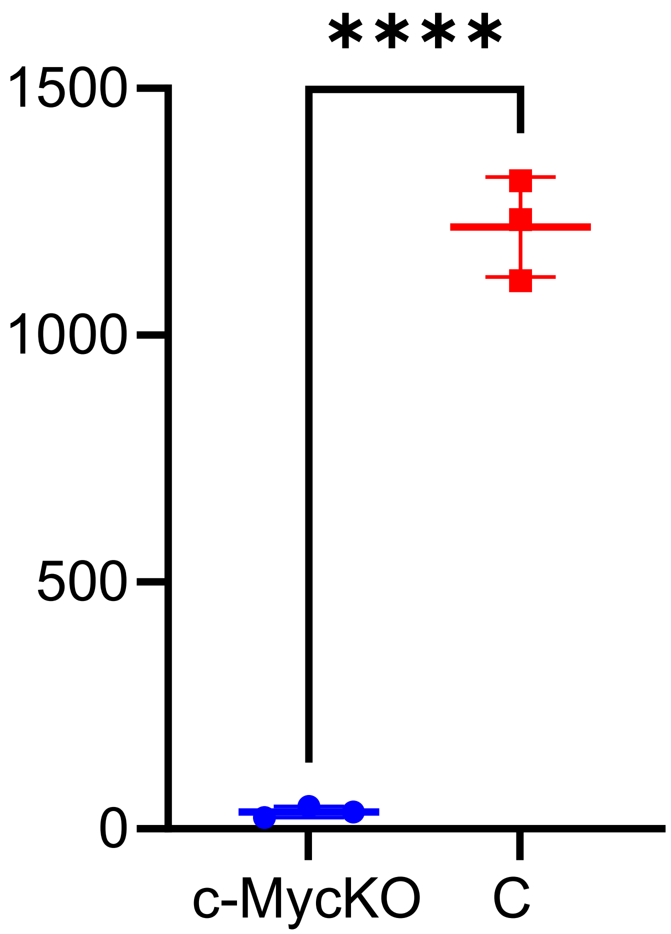

Supplementary Fig S2. Relative gene expression fold of c-Myc in c-Myc knockout and control groups, with respect to untreated groups using qPCR confirmed the successful knockout of c-Myc in the knockout group. C: control group, c-MycKO: c-Myc knockout group. ****: $P \leq 0.001$.

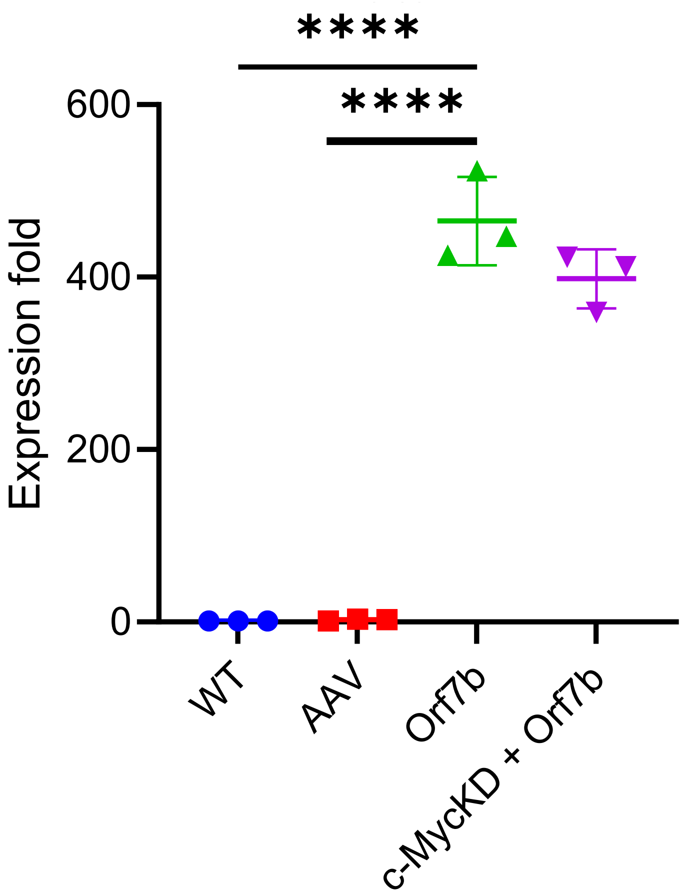

Supplementary Fig S3. Relative gene expression fold of Orf7b in wild type, control, Orf7b overexpressed and c-Myc knockdown Orf7b overexpressed groups, with respect to wild type group using qPCR confirmed the successful overexpression of Orf7b in the Orf7b and c-MycKD +Orf7b groups. WT: wild type group, AAV: control group, Orf7b-Orf7b overexpressed group, c-MycKD + O7b: c-Myc knockdown group overexpressed with Orf7b. ****: $P \leq 0.001$.
